# Spatiotemporal variability in surface energy balance across tundra, snow and ice in Greenland

**DOI:** 10.1007/s13280-016-0867-5

**Published:** 2017-01-23

**Authors:** Magnus Lund, Christian Stiegler, Jakob Abermann, Michele Citterio, Birger U. Hansen, Dirk van As

**Affiliations:** 1 Department of Bioscience, Arctic Research Centre, Aarhus University, Frederiksborgvej 399, 4000 Roskilde, Denmark; 2 Asiaq, Greenland Survey, Qatserisut 8, 3900 Nuuk, Greenland; 3 Geological Survey of Denmark and Greenland, Øster Voldgade 10, 1350 Copenhagen, Denmark; 4 Department of Geosciences and Natural Resource Management, Center for Permafrost (CENPERM), University of Copenhagen, Øster Voldgade 10, 1350 Copenhagen K, Denmark

**Keywords:** Glacier, Ice sheet, Permafrost, Surface energy balance, Tundra

## Abstract

The surface energy balance (SEB) is essential for understanding the coupled cryosphere–atmosphere system in the Arctic. In this study, we investigate the spatiotemporal variability in SEB across tundra, snow and ice. During the snow-free period, the main energy sink for ice sites is surface melt. For tundra, energy is used for sensible and latent heat flux and soil heat flux leading to permafrost thaw. Longer snow-free period increases melting of the Greenland Ice Sheet and glaciers and may promote tundra permafrost thaw. During winter, clouds have a warming effect across surface types whereas during summer clouds have a cooling effect over tundra and a warming effect over ice, reflecting the spatial variation in albedo. The complex interactions between factors affecting SEB across surface types remain a challenge for understanding current and future conditions. Extended monitoring activities coupled with modelling efforts are essential for assessing the impact of warming in the Arctic.

**Electronic supplementary material:**

The online version of this article (doi:10.1007/s13280-016-0867-5) contains supplementary material, which is available to authorized users.

## Introduction

In the Arctic, atmospheric warming due to increases in greenhouse gases has been approximately twice as high compared with the global average (Graversen et al. 2008). This is due to a number of feedback processes, many of which are related to the surface energy balance (SEB). In Greenland, the last decade was reportedly the warmest since the onset of Greenland meteorological measurements, with surface air temperature (i.e. at ca. 2 m above the surface) for the period 1993–2010 showing a linear trend of 0.16 °C year^−1^ (Masson-Delmotte et al. 2012). By the end of the twenty-first century, climate models predict average arctic land area surface air temperature to exceed late twentieth century temperatures by 4.2 °C (Collins et al. 2013). Greenland air temperature for the end of the twenty-first century is estimated to be 3.3 ± 1.3 °C warmer compared with late twenty-first century, with most pronounced warming during winter (Stendel et al. 2007; Franco et al. 2011; Masson-Delmotte et al. 2012). Precipitation is expected to increase by more than 50% in the Arctic regions, mainly driven by increased poleward transport and intensified local moisture exchange (Collins et al. 2013).

The Greenland ice sheet (GrIS), which is the largest freshwater reservoir in the northern hemisphere, experienced accelerated mass loss over the past decades, as a result of atmospheric warming and darkening of the ice sheet (Box et al. 2012). The average GrIS mass loss during the period 1991–2015 is estimated to equal ~0.47 mm year^−1^ of global sea level rise (van den Broeke et al. 2016). Future projections of GrIS mass balance suggest strong exponential relationship between GrIS surface melt and surface air temperature (Franco et al. 2013); with approx. 2.5 times higher surface runoff by 2070–2080 compared with 1950–1959 and an average sea level rise of 4–9 cm by the end of the twenty-first century (Fettweis et al. 2013). Greenland glaciers and ice caps independent from the GrIS further contribute to the total mass loss and, owing to their higher climate sensitivity, they have been found to account for 14–20% of the overall 2003–2008 mass loss from Greenland (Bolch et al. 2013).

Permafrost (perennially frozen ground) and the annual freeze and thaw cycles of the above-lying active layer are other important components in the Arctic climate system. Permafrost thermal state and active layer development are closely linked with physical and biological processes such as surface hydrology, geomorphology and biogeochemical cycling. Permafrost soils contain approx. 50% of the global belowground organic carbon (Tarnocai et al. 2009), and its partial release through carbon dioxide and methane emissions to the atmosphere may significantly amplify warming (Schaefer et al. 2014). During the twentieth century, climate-driven increase in air temperature has led to higher ground surface and permafrost temperatures, increased permafrost degradation and thicker active layers (Romanovsky et al. 2010).

The overall driver of ecosystem functioning, permafrost thermal state and glacier surface mass balance is the energy exchange at the Earth’s surface. The radiative, turbulent and conductive heat fluxes between the atmospheric boundary layer and the Earth’s surface are in balance as expressed by the SEB. Changes in magnitude and partitioning of SEB components may feedback on climate change, and as such the SEB of Arctic environments are attracting increased attention. Nevertheless, direct observations of SEB in the Arctic are scarce, contributing to the lack of comparative studies between glacier ice and tundra ecosystems. This limited knowledge results in incomplete understanding of land–atmosphere energy exchange in the Arctic and its connection to the global climate system. Quantifying the spatiotemporal variability in the magnitude and partitioning of SEB components is a key for improved understanding of the coupled cryosphere–atmosphere system in the large Arctic region. Therefore, in this paper, we synthesize SEB components and key controlling factors across Greenlandic terrestrial environments. The main purpose is to study SEB for the dominant surface types in Greenland, including wet and dry tundra, snow and ice. This will be pursued through an investigation of how variations in key variables, including surface type, albedo, snow and cloudiness, affect SEB components.

## Materials and methods

### Site description

**Fig. 1:**
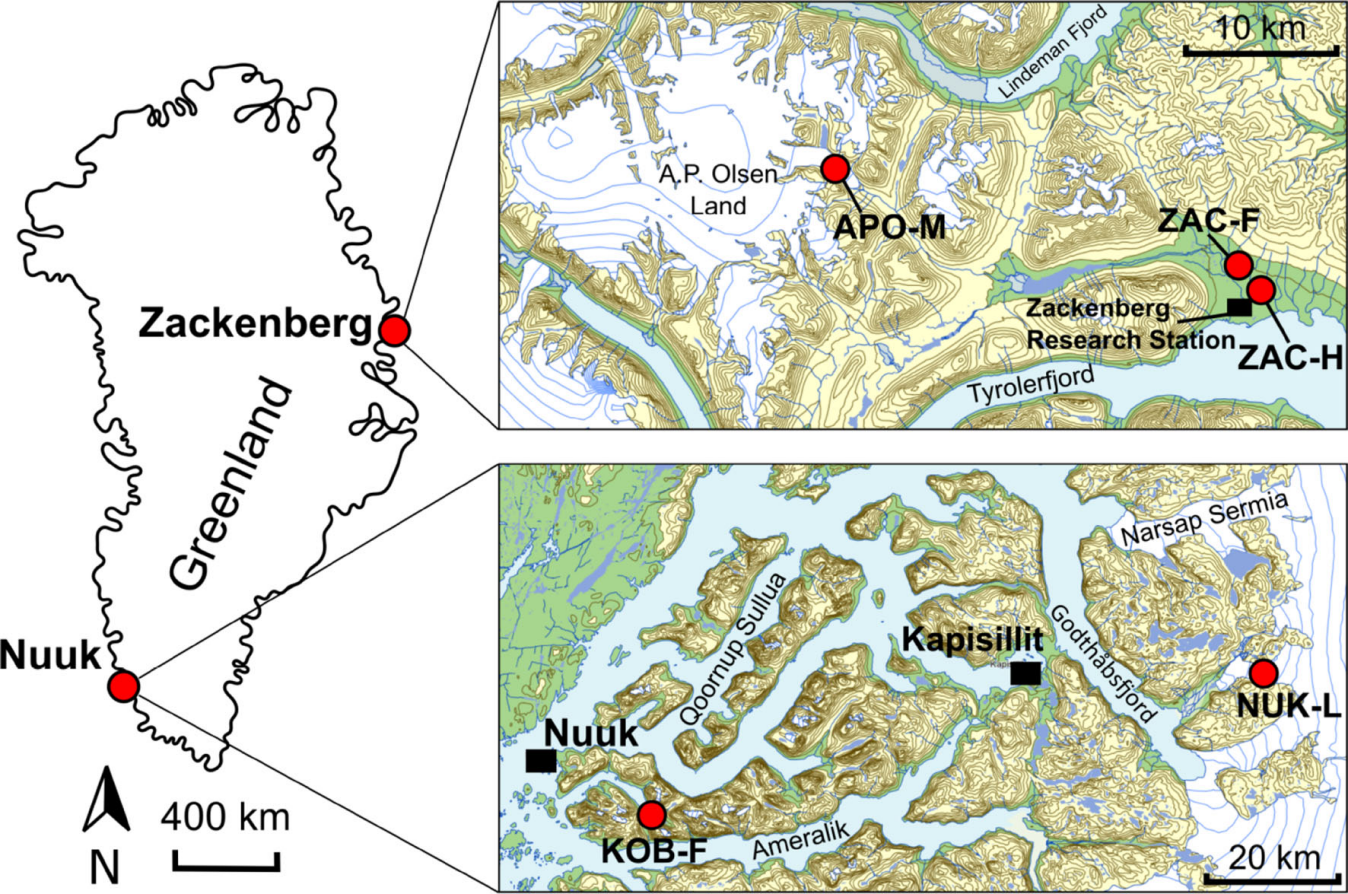
Map of Greenland with inserts showing the Zackenberg and Nuuk regions and the associated monitoring sites (Map source: NunaGIS)

Observations from five measurement sites during 2012–2015 are included in this study (Fig. 1; Table 1). Three sites are located in northeast Greenland near the Zackenberg Research Station: dry tundra heath (ZAC-H), wet tundra fen (ZAC-F) and the southeastern outlet glacier of the A.P. Olsen ice cap (APO-M). Two sites are located in southwest Greenland: wet tundra fen in Kobbefjord (KOB-F) and the lower ice sheet area east of Nuuk (NUK-L).

**Table 1:** Information on surface type, location, data and citation for the measurement sites

Site name	Surface type	Latitude (°N)	Longitude (°W)	Elevation (m)	Data years	Primary citation
APO-M	Glacier ablation area	74.625	21.376	660	2012–2013	Citterio et al. (2010)
ZAC-F	Wet tundra (fen)	74.481	20.555	40	2012–2014	Stiegler et al. (2016)
ZAC-H	Dry tundra (heath)	74.473	20.550	40	2012–2014	Lund et al. (2012)
NUK-L	Ice sheet ablation area	64.482	49.533	540	2014–2015	Van As et al. (2014)
KOB-F	Wet tundra (fen)	64.131	51.386	50	2013–2015	Westergaard-Nielsen et al. (2013)

The Zackenberg region is located in the high Arctic within the continuous permafrost zone. The 1996–2013 mean annual temperature for the Zackenberg Valley is −8.9 °C, with July being the warmest month (6.2 °C) and January the coldest (−22.4 °C). Mean annual precipitation sum is 270 mm, of which ca. 85% falls as snow (Hansen et al. 2008). The area has polar night 6 November to 4 February and polar day 1 May to 12 August. The fen (ZAC-F) is dominated by sedges such as *Eriophorum scheuchzeri*, *Carex stans* and *Duponita psilosantha,* while the heath (ZAC-H) is dominated by *Cassiope tetragona, Dryas integrifolia* and *Vaccinium uliginosum.* The A.P. Olsen ice cap is located approximately 35 km northwest of the Zackenberg Valley. The weather station APO-M is positioned in the ablation zone with bare ice surface in the melt season. Summertime (June–August) temperatures are on average 1.6 °C lower compared with the valley bottom, whereas wintertime (December–February) temperatures are 2.4 °C higher.

The Nuuk region is located in the low Arctic. Long-term (1961–1990) mean annual temperature and precipitation sum are −1.4 °C and 750 mm, respectively, with March being the coldest month (−8.0 °C) and July the warmest (6.5 °C; Cappelen 2012). The Kobbefjord valley is located ca. 20 km southeast of the Greenlandic capital Nuuk. The wet tundra fen (KOB-F) is located within the Kobbefjord valley; an area which is devoid of permafrost. Vegetation is dominated by *E. angustifolium* and *Scirpus caespitosus*. The lower ice sheet weather station NUK-L is situated in the ablation zone of the GrIS, ca. 100 km northeast of Kobbefjord. The region is located south of the polar circle and thus experiences a more pronounced seasonality than Zackenberg. The ice station is colder than the tundra site both during summer (−3.9 °C) and winter (−3.5 °C).

### Monitoring data

All sites are equipped with four component radiometers and standard meteorological equipment for measurements of air temperature and relative humidity, wind speed and direction, barometric pressure, snow depth and subsurface temperature (Table 2). The glacier (APO-M) and ice sheet (NUK-L) sites are equipped with inclinometers used to correct the radiation components for tilt as they are placed on a glacier surface in the ablation area where surface changes considerably throughout the year (Citterio et al. 2015). The tundra stations sites are equipped with eddy covariance instrumentations for direct measurements of sensible and latent heat fluxes (Table 2). Subsurface heat flux is measured just below the tundra soil using heat flux plates (Table 2).

**Table 2:** Instrumentation used at the measurement sites. Measurement height/depth in metres when snow cover is absent is indicated in parentheses

	APO-M	ZAC-F	ZAC-H	NUK-L	KOB-F
Meteorology					
Radiometer	Kipp & Zonen CNR1 (3)	Kipp & Zonen CNR4 (4)	Kipp & Zonen CNR1 & CNR4 (4)	Kipp & Zonen CNR1 (3)	Kipp & Zonen CNR4 (3)
Air temperature/relative humidity	Rotronic MP100 (3)	Campbell Sci. CS215 (3)	Vaisala HMP 45D (2)	Rotronic MP100 (3)	Vaisala HMP 45D (2)
Wind speed	R.M. Young 05103-5 (3)	Gill HS-50 (3)	Gill R3-50 (3)	R.M. Young 05103-5 (3)	Gill R3-50 (2)
Wind direction	R.M. Young 05103-5 (3)	Gill HS-50 (3)	Gill R3-50 (3)	R.M. Young 05103-5 (3)	Gill R3-50 (2)
Barometric pressure	Setra 278 (1)	Setra 278 (1)	Vaisala PTB101B (1)	Setra 278 (1)	Setra 278 (2)
Snow depth	Campbell Sci. SR50a (3)	Campbell Sci. SR50a (4)	Campbell Sci. SR50a (4)	Campbell Sci. SR50a (3)	Campbell Sci. SR50a (3)
Subsurface temperature	Thermistors (−1, −2, −3, −4, −5, −6, −7, −8)	Thermistors (−0.02, −0.1, −0.5)	Thermistors (−0.02, −0.1, −0.4)	Thermistors (−1, −2, −3, −4, −5, −6, −7, −10)	Thermistors (−0.05, −0.1, −0.5)
Ground heat flux	–	Hukseflux HFP01 (−0.05)	Hukseflux HFP01 (−0.05)	–	Hukseflux HFP01 (−0.05)
Eddy covariance					
3D sonic anemometer	–	Gill HS-50 (3)	Gill R3-50 (3)	–	Gill R3-50 (2)
H_2_O gas analyser	–	LI-COR 7200 (3)	LI-COR 7000 (3)	–	LI-COR 7000 (2)

### Data analyses

The radiation balance equation can be written as:

1 $$ R_{\text{n}} = {\text{SW}}_{\text{in}} + {\text{SW}}_{\text{out}} + {\text{LW}}_{\text{in}} + {\text{LW}}_{\text{out}} = {\text{SW}}_{\text{net}} + {\text{LW}}_{\text{net}} , $$

where *R*_n_ is the net radiation, SW_in_, SW_out_ and SW_net_ are the incoming, outgoing and net shortwave components and LW_in_, LW_out_ and LW_net_ are the incoming, outgoing and net longwave components, respectively.

The energy balance at the surface-atmosphere interface is:

2 $$ R_{\text{n}} + H + {\text{LE}} + G + M = 0, $$

where *H* is the sensible heat flux, LE the latent heat flux, *G* the ground heat flux (vertical transfer of heat through snow, soil or ice) and *M* the energy used for melting snow and ice at the surface. All fluxes are defined positive when adding energy to the surface.

For the tundra sites sensible and latent heat fluxes were calculated based on standard eddy covariance community methodology (Aubinet et al. 2000) using EdiRe (ZAC-H, KOB-F) and EddyPro (ZAC-F) software packages: including despiking, 2D coordinate rotation, time lag removal by covariance optimization, block averaging, frequency response correction and Webb–Pearman–Leuning correction. Both sensible and latent heat fluxes were gap-filled using a look-up table approach. The energy balance closure during the study period was on average 85, 81 and 83% for ZAC-H, ZAC-F and KOB-F, respectively. More information on the eddy covariance systems, processing and uncertainties is provided by Lund et al. (2012, 2014) and Stiegler et al. (2016). Heat stored above the soil heat flux plates was calculated according to Lund et al. (2014) and added to the measured flux. During the snow-covered period, snow-pack temperature measurements were used to calculate heat storage in the snow pack.

An SEB model (Van As 2011, 2012) was used to quantify the components of the SEB (Eq. 2) for the ice sites (APO-M, NUK-L), based on the meteorological observations. Sensible and latent heat fluxes were calculated using the bulk method assuming Monin–Obukhov similarity. The calculations make use of near-surface gradients in wind speed, temperature and specific humidity using the surface as the lower level (Van As 2011). The surface roughness length for momentum for ice and snow was set to 1 × 10^−3^ m and 1 × 10^−4^ m, respectively (Brock et al. 2006). Calculations of sensible, latent and ground heat flux make use of the variable of surface temperature, for which the equation can be solved iteratively. Melt energy is produced when the SEB components cannot be balanced, which occurs when the surface temperature is limited by the melting point. For more model details, see Van As (2011) and references therein.

Albedo was calculated as “accumulated albedo” following van den Broeke et al. (2004), defined as the ratio of accumulated values of incoming and outgoing shortwave radiation within a 24-h sliding time window centred on the moment of observation. Only observations during solar elevation angles above 20° were used in the calculations. Cloud cover was approximated based on the strong relationship between incoming longwave radiation and surface air temperature, making use of the temperature dependencies of clear-sky longwave radiation and overcast black-body radiation (Van As et al. 2005; Van As 2011). A cloud cover fraction was calculated from linear interpolation of these dependencies for a given pair of incoming longwave radiation and surface air temperature observations. Clear sky was defined as having a cloud cover fraction below 0.15, while overcast conditions were assumed when cloud cover fraction exceeded 0.85. The cloud radiative effect (CRE) was calculated as the difference in net radiation between all-sky and clear-sky conditions (Van Tricht et al. 2016).

## Results and discussion

### Radiation budget

**Fig. 2:**
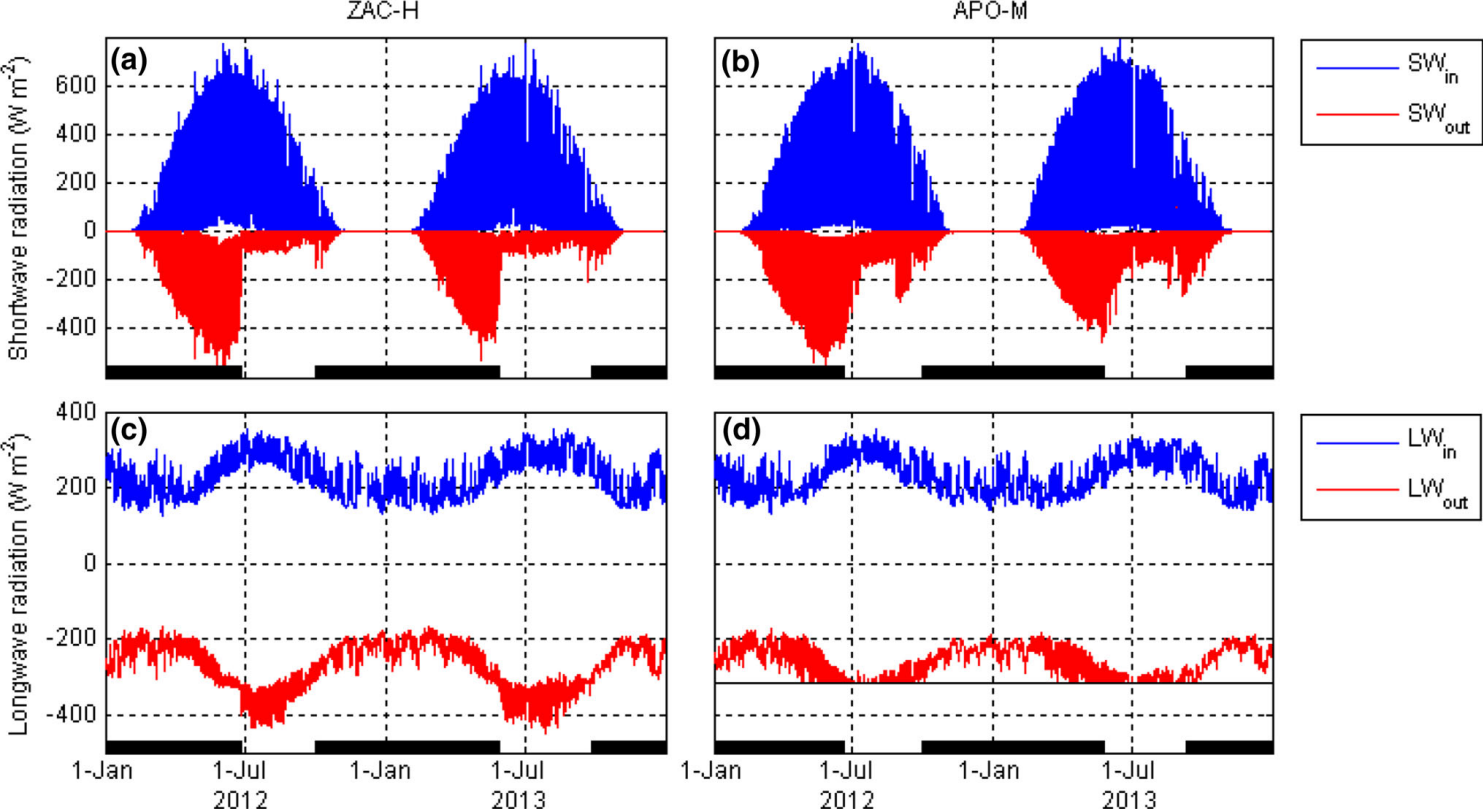
Incoming (SW_in_) and outgoing (SW_out_) shortwave radiation (**a**, **b**) and incoming (LW_in_) and outgoing (LW_out_) radiation (**c**, **d**) at Zackenberg dry heath tundra (ZAC-H; **a**, **c**) and A.P. Olsen glacier (APO-M; **b**, **d**) during 2012–2013. *Thick black lines* in the *bottom* of the panels indicate periods with continuous snow cover. *Thin black line* in **d** indicates outgoing longwave radiation at the melting point of ice (−316 W m^−2^)

During the snow-free period, tundra sites reflected less shortwave radiation compared with ice sites due to their surfaces being darker (Fig. 2). At the same time, there was more outgoing longwave radiation at tundra sites because of higher ground surface temperature. Tundra surfaces may heat up to 20–30 °C during summer, while ice surfaces are naturally restricted to the melting point, which limits ice sheet and glacier outgoing longwave radiation to 316 W m^−2^. The lower amount of shortwave radiation absorbed at ice surfaces is more than compensated for by smaller longwave losses, resulting in more available surface net radiation at ice surfaces compared with tundra surfaces. For example, mid-July to mid-August mean net radiation at APO-M was 151 and 147 W m^−2^ in 2012 and 2013, respectively, compared with 111 and 112 W m^−2^ at ZAC-H and 114 and 113 W m^−2^ at ZAC-F for corresponding periods.

Absorbed solar radiation is the primary, present-day energy source for Greenland glacier ice melt on an annual basis (van den Broeke et al. 2008; Box et al. 2012; van As et al. 2012), although non-radiative energy fluxes can dominate during shorter periods (Fausto et al. 2016). Net shortwave radiation is also projected to explain most of the melt increase in the future, mainly due to the positive surface albedo feedback (Box et al. 2012; Franco et al. 2013). For tundra, shortwave radiation is the dominant energy source during the snow-free period, controlling the magnitude of other SEB components as well as the thermal state of permafrost (Westermann et al. 2009; Langer et al. 2011; Lund et al. 2014).

**Fig. 3:**
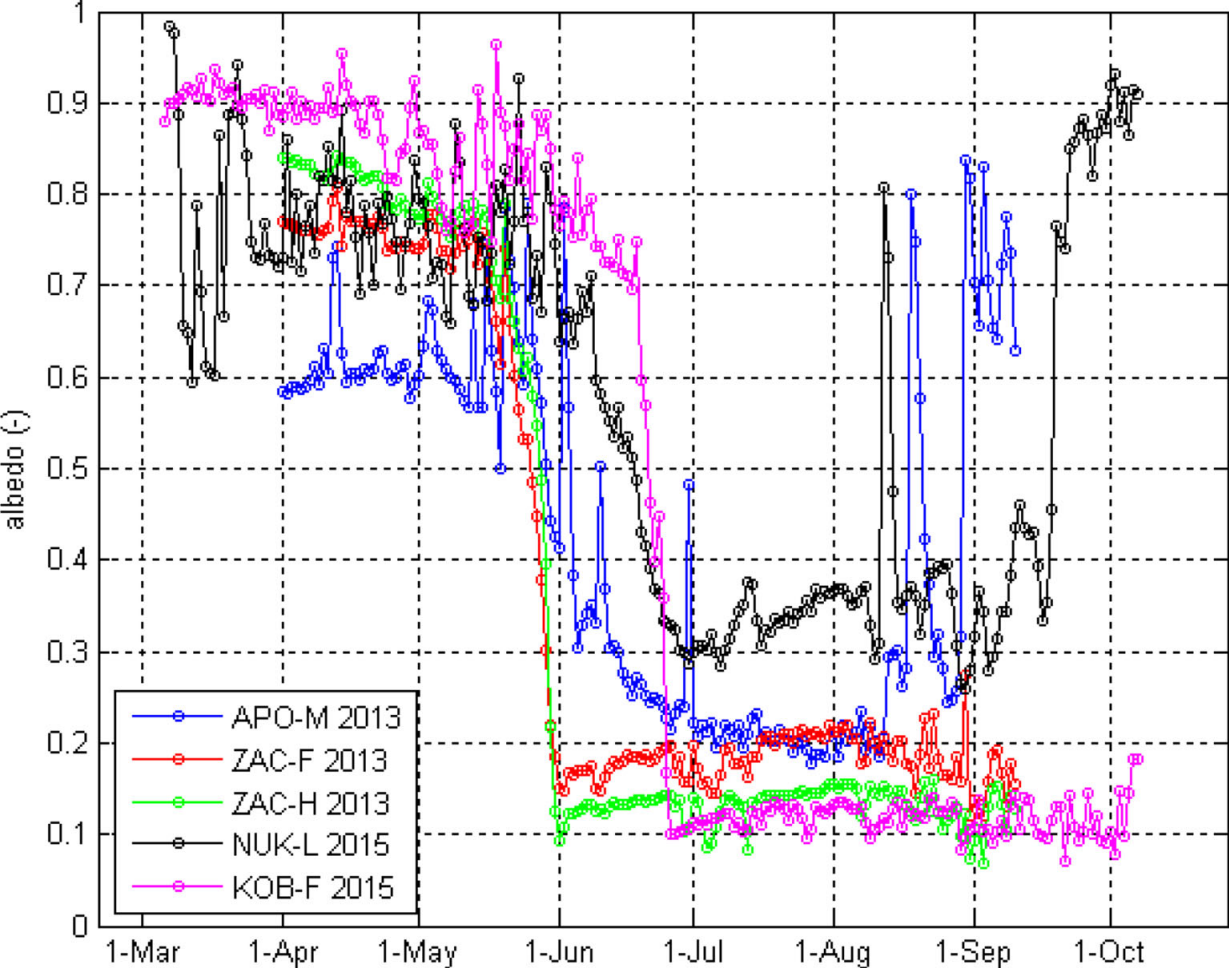
Daily means of accumulated albedo (van den Broeke et al. 2004) for the Zackenberg sites (APO-M, ZAC-F, ZAC-H) in 2013 and the Nuuk sites (NUK-L, KOB-F) in 2015

During the snow-covered period, differences in radiation budget components among sites were small, as a continuous snow cover results in similar surface characteristics irrespective of underlying surface type. Differences in net shortwave radiation and albedo are mainly driven by the capacity of the snow pack to cover the underlying surface, including its microtopography and surface elements, e.g. vegetation (Fig. 3). A thin snow cover, e.g. at APO-M during early 2013 (maximum snow depths <0.2 m) with ice partly exposed in the immediate surroundings of the weather station, may result in lower albedo and higher net shortwave radiation compared with completely snow-covered surfaces (Stiegler et al. 2016).

The disappearance of snow at tundra surfaces marked a distinct drop in surface albedo within a few days, while for the glacier and ice sheet sites, the decrease was less pronounced (Fig. 3). During snow melt, albedo of ZAC-F decreased slightly earlier than ZAC-H due to higher and denser vegetation that is exposed earlier, increasing surface heat absorption and snow melt. The timing of snow melt has a strong influence on albedo across all surface types; during 2013, the snow melt period ended early for the Zackenberg tundra sites (around 31 May), while for KOB-F in 2015, it ended late (25 June). During the snow-free period, albedo across the sites generally ranked NUK-L > APO-M > ZAC-F > ZAC-H > KOB-F. Albedo at the ice sheet site (NUK-L) rarely dropped below 0.3, whereas for the glacier site (APO-M), albedo reached just below 0.2. Albedo at the tundra sites generally ranged between 0.1 and 0.2, which is within the range reported for a number of tundra sites (Eugster et al. 2000).

There was an increasing trend in albedo for the first two-thirds of the snow-free period for the tundra sites, followed by a decrease towards the onset of the snow cover in autumn. The increase in albedo may be associated with decreasing soil wetness and increasing vegetation biomass covering darker soils (Eugster et al. 2000; Lund et al. 2014). Sudden drops in snow-free period albedo for tundra are associated with precipitation events wetting the soils. On 30 June 2013, there was a decrease in albedo for ZAC-F and ZAC-H but an increase for APO-M, because of rainfall in the valley and snowfall on the glacier. Differences in snow-free albedo within tundra sites are related to wetness and vegetation composition. The ZAC-F site is characterized by a relatively bright patch of sedges compared with a darker and sparser shrub cover in the ZAC-H site (Fig. S1). The wet tundra site in Kobbefjord (KOB-F) is also dominated by sedges, however, of less stature compared with ZAC-F exposing more bare, wet and darker soil. Furthermore, the higher solar angles at KOB-F compared with Zackenberg lead to relatively lower albedo values.

For ice surfaces, albedo may decrease during the snow-free period due to impurities associated with dust deposition or algal growth (Bøggild et al. 2010; Uetake et al. 2010), which could be the case for APO-M in 2013. For both ice sites, snowfall events in August temporarily increased albedo to ca. 0.8.

### Surface energy balance

**Fig. 4:**
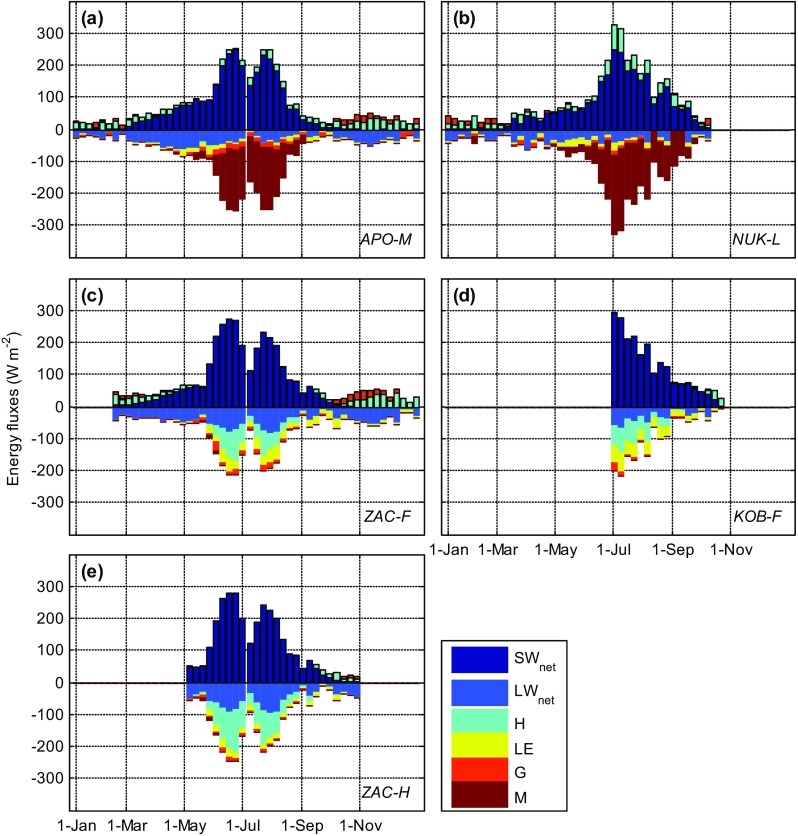
Weekly means of SEB components (Eq. 2) in 2013 from Zackenberg sites, **a** APO-M, **c** ZAC-F, **e** ZAC-H; and in 2015 from Nuuk sites, **b** NUK-L, **d** KOB-F. Only periods when all components of the surface energy balance were available are shown

The annual evolution of SEB components reveals fundamental differences between tundra and ice sites (Fig. 4). Both net shortwave and net longwave radiation had higher amplitudes during the snow-free season at the tundra sites, associated with aforementioned differences in albedo and ground surface temperature. During summer, melt was the major energy sink for the glacier and ice sheet sites, with relatively low energy losses through ground heat and latent heat flux. Sensible heat flux was positive since air temperature exceeded ground surface temperature (max. 0 °C), making sensible heat an important energy source for ice-covered surfaces experiencing melt (Braithwaite 1981; Ohmura 1982).

On the contrary, for tundra surfaces, sensible heat flux was generally negative due to high ground surface temperature exceeding that of the air (Langer et al. 2011). Higher surface temperature compared with ice-covered surfaces also resulted in higher latent heat flux, which together with sensible heat flux and net longwave radiation constituted the most important energy sinks. Wet tundra sites generally have a relatively larger energy partitioning into latent heat compared with sensible heat (Eugster et al. 2000), i.e. the Bowen ratio (H/LE) was lower for wet sites (ZAC-F and KOB-F in this study) compared with dry sites (ZAC-H). The relatively low latent heat flux at ZAC-H during 2013 was related to moisture-limited soil conditions caused by low snow accumulation during preceding winter (Stiegler et al. 2016). Furthermore, the Zackenberg area is characterized by onshore winds during summer, carrying cold, moisture-laden air masses over land suppressing latent heat flux and enhancing sensible heat flux (Lund et al. 2014).

Ground heat flux is an important SEB component for permafrost soils. Energy sunk by the ground heat flux is used to heat the soil and thaw the active layer (Rouse 1984). In ZAC-F and ZAC-H, ground heat flux consumed 5–30% of net radiation during the snow-free season (Lund et al. 2014; Stiegler et al. 2016), a ratio that has decreased since 2000 in association with increased active layer depth (Lund et al. 2014). At KOB-F, which is devoid of permafrost, ground heat flux consumed <5% during the snow-free period. Tundra ground heat flux is generally higher immediately after snow melt due to higher soil conductivity associated with high soil water content as well as a steep temperature gradient between the surface and permafrost table (Rouse 1984). As the season progresses, there was a general drying trend, which in combination with thicker active layers results in decreasing ground heat flux (Westermann et al. 2009; Lund et al. 2014). Since permafrost is a heat sink reducing surface temperature and thus turbulent heat fluxes, increased active layer depths act to amplify climatic warming (Lund et al. 2014).

Similar to the radiation components, differences in sensible, latent and ground heat flux across sites were small during the snow-covered period. During the winter period, net radiation was dominated by negative net longwave radiation due to low or completely absent shortwave radiation. The radiative losses were balanced by a positive sensible heat flux, providing energy to the surface and cooling the atmospheric boundary layer, and a positive ground heat flux, indicating cooling of the subsurface. Wintertime losses of latent heat through sublimation were generally small; however, latent heat flux can contribute more on shorter (synoptic) time scales, e.g. associated with increased wind speed (Westermann et al. 2009; van As 2011).

### Importance of snow

**Fig. 5:**
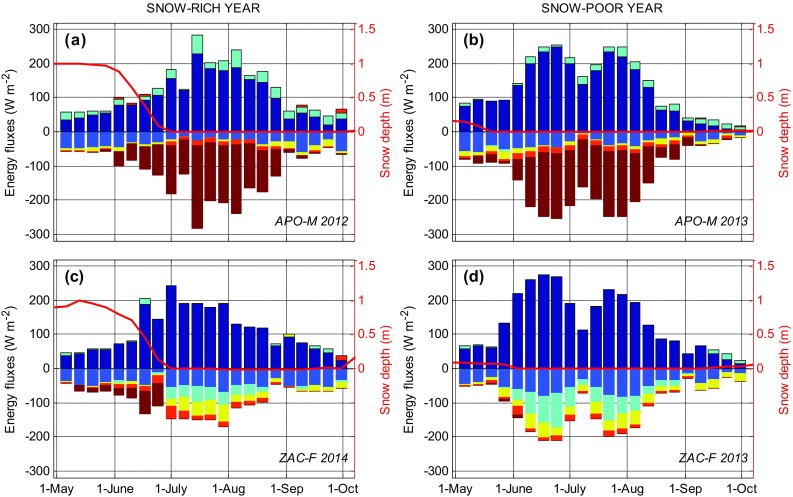
Weekly means of SEB components (Eq. 2) for APO-M (**a**, **b**) and ZAC-F (**c**, **d**) during May–October in a snow-rich year (**a**, **c**) and a snow-poor year (**b**, **d**), respectively. For legend, see Fig. 4. *Red line* indicates snow depth

The importance of snow for SEB is exemplified by comparing a snow-rich year (2012 in APO-M, 2014 in ZAC-F) and a snow-poor year (2013; Fig. 5). A thick snow pack insulates the ground/ice from low wintertime air temperature and large energy losses. The ground thermal regime is highly sensitive to the timing and length of seasonal snow cover (Callaghan et al. 2012). The timing of spring snow melt and associated changes in albedo is of key importance for the SEB, since it coincides with maximum inputs of solar radiation. Once snow-free, sensible heat flux at tundra surfaces shifts sign and constitutes an energy sink during most of the snow-free season. At the on-ice sites, a short snow-free period decreases the period of ice exposure and generally results in lower annual net ablation values (Box et al. 2012; Fausto et al. 2016). In the case of APO-M, energy consumed by melt during the snow-free period totalled 850 and 1070 MJ m^−2^ for a snow-rich (2012) and a snow-poor year (2013), respectively.

The picture is similar for a tundra site with more net radiation during a snow-poor year; however, the additional energy absorbed by the surface is consumed by sensible and latent heat flux and possibly also ground heat flux. For the former, the partitioning between sensible and latent heat flux (Bowen ratio) is dependent on surface wetness (Eugster et al. 2000). Snow-poor years with long snow-free periods generally result in low soil moisture content, especially in Zackenberg where a majority of the precipitation falls as snow. Snow-poor years are therefore generally characterized by high Bowen ratios (Lund et al. 2014; Stiegler et al. 2016), resulting in a larger fraction of sensible heat flux warming the atmospheric boundary layer during dry summers. For ZAC-F, the mean snow-free period Bowen ratios were 1.67 and 0.99 for a snow-poor (2013) and a snow-rich year (2014), respectively. The corresponding Bowen ratios for ZAC-H were 3.70 and 1.24, respectively.

Soil heat flux, warming of the active layer and permafrost thaw are also dependent on soil moisture content. More energy is needed to thaw wet soils resulting in shallower thaw depths in wet sites. However, once thawed, a higher soil moisture content results in higher soil thermal conductivity and more efficient heat transfer into the soil column. Therefore, the limiting effect of dry soils on soil heat flux may counteract the effect of more energy absorbed at the surface during snow-poor years with long snow-free period. At a CALM (Circumpolar Active Layer Monitoring) field in the Zackenberg valley close to the ZAC-H site, the maximum thaw depth was similar in 2013 and 2014 (−0.738 and −0.743 m, respectively), despite 27 days earlier snow melt in 2013.

In Greenland, climate models generally predict a decrease in snow accumulation and in length of snow cover for southern Greenland, while in northern Greenland, models predict a deeper snow cover, lengthening of the melting season and increased variability in thaw periods during winter (Stendel et al. 2007; Masson-Delmotte et al. 2012). An earlier loss of snow cover on GrIS and glaciers promotes surface melt as bare ice areas with lower albedo compared with snow are exposed to direct radiation (Franco et al. 2013). This additional input of radiation may be of higher importance to surface changes than atmospheric warming (Charalampidis et al. 2015). In tundra, an earlier loss of the insulating effect of snow may promote permafrost thaw due to increased heat exchange (Callaghan et al. 2012). Continued permafrost thaw will trigger hydrological changes that, dependent on hydrological regime, can result in some areas getting wetter while others may get drier (Bring et al. 2016).

### Importance of clouds

Clouds play an important role in modulating Earth’s radiative fluxes. Overcast conditions limit incoming shortwave radiation, while at the same time, incoming longwave radiation is increased. Variation in cloudiness is responsible for the rapid fluctuations in the radiation component time series (Fig. 2). Previous studies report that the GrIS and Arctic tundra are sensitive to cloud cover type and changes in cloud cover (van den Broeke et al. 2008; Westermann et al. 2009; Bennartz et al. 2013; Van Tricht et al. 2016). Clouds have been found to generally have a positive radiative effect over the course of a year across the GrIS (i.e. the radiation paradox; Ambach 1974), since the positive longwave cloud effect exceeds the negative shortwave effect. The total cloud radiative effect is dependent on cloud optical properties, which in turn is related to cloud ice and water content (van den Broeke et al. 2008; Bennartz et al. 2013).

**Fig. 6:**
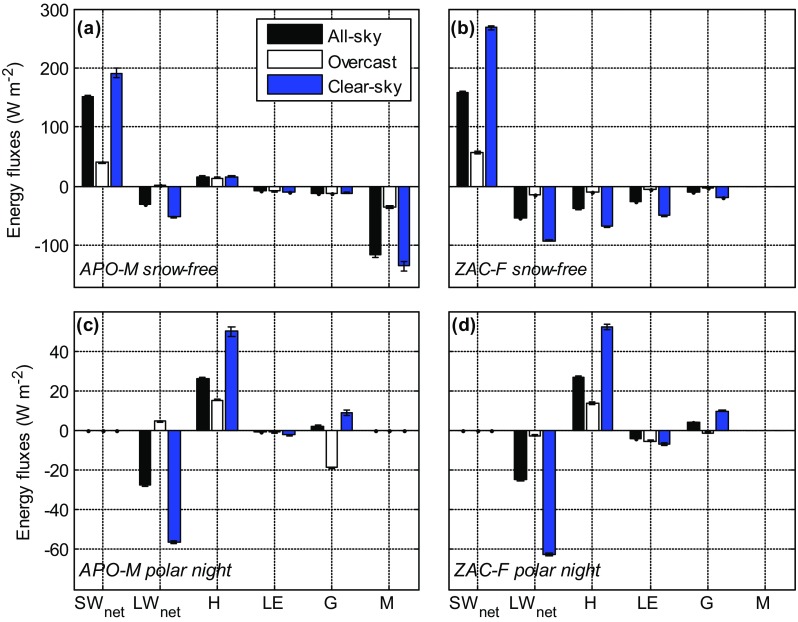
SEB component means derived from all-sky, overcast and clear-sky conditions, respectively, during snow-free (**a**, **b**) and polar night (**c**, **d**) periods at APO-M (**a**, **c**) and ZAC-F (**b**, **d**). *Error bars* indicate standard error

During polar night, the SEB components at APO-M and ZAC-F showed a similar behaviour (Figs. 4, 6) due to similar longwave characteristics of the snow-covered surfaces. Net longwave radiation was negative for all-sky and clear-sky conditions, while it was close to zero for overcast conditions. Cloud radiative effect was positive for all sites during the dark winter period, on average 28.6 W m^−2^ (Table 3), indicating net cloud warming. Sensible heat flux was higher for both surface types during clear-sky conditions, partly compensating for increased longwave losses.

**Table 3:** Cloud radiative effect (CRE) based on hourly (APO-M, NUK-L) and half-hourly (ZAC-F, ZAC-H, KOB-F) observations. Values in parentheses represent number of clear-sky observations

Years	Period	APO-M	ZAC-F	ZAC-H	NUK-L	KOB-F
2012	Snow-free^a^	0.1 (266)	−41.1 (1296)	−39.9 (1231)	n/a	−123.7 (565)
2012/13	Winter^b^	13.6 (32)	19.2 (214)	23.5 (214)	n/a	26.9 (419)
2013	Snow-free	−4.0 (358)	−63.1 (1494)	−59.1 (1494)	n/a	−96.7 (547)
2013/14	Winter	29.0 (99)	37.7 (326)	35.0 (326)	28.3 (180)	34.4 (322)
2014	Snow-free	n/a	−52.8 (733)	−57.6 (732)	11.4 (152)	−122.8 (888)
2014/15	Winter	n/a	n/a	n/a	31.2 (65)	36.2 (197)
2015	Snow-free	n/a	n/a	n/a	24.8 (252)	−151.2 (730)

^a^Snow-free period here starts when surface is snow-free (earliest 1 June) and ends when surface is snow-covered (latest 31 August)

^b^Winter period here refers to 6 November–4 February, i.e. the period of polar night in Zackenberg region

During the snow-free period, clouds modulate the SEB components in a similar manner for both ice and tundra sites. Overcast conditions resulted in a decrease in net shortwave radiation by approx. 80% compared with clear-sky conditions (Fig. 6). At ZAC-F, net longwave radiation remained an energy sink across sky conditions. However, at APO-M, net longwave radiation was an energy sink for all-sky and clear-sky conditions, while for overcast conditions it became a small energy source to the surface, which can result from relatively low outgoing longwave radiation due to low ground surface temperature. Negative shortwave cloud effect exceeded positive longwave effect for the tundra sites resulting in a negative cloud radiative effect (Table 3). For NUK-L, clouds had a warming effect also during summer, while for APO-M, cloud radiative effect was close to zero. These differences reflect the spatial variation in albedo across surface types (Fig. 3).

The differences in the radiative components due to variations in cloudiness had no pronounced impact on the sign and magnitude of sensible, latent and ground heat fluxes at APO-M. However, the energy consumed by melt was ca. four times higher for clear-sky compared with overcast conditions. For the tundra site, there was a large difference in turbulent heat fluxes during overcast vs. clear-sky conditions, with sensible and latent heat flux being ca. seven and eight times higher, respectively, for clear-sky conditions. Clear-sky conditions resulted in high ground surface temperature increasing turbulent heat losses. Latent and ground heat flux showed similar intensities during overcast conditions, while during all-sky and clear-sky conditions, latent heat flux dominated over ground heat flux.

The cloud cover calculations in this study are simplified and do not separate between cloud types, water and ice content or optical thickness. Also, due to the usage of incoming longwave radiation in the cloud cover classification, differences in net longwave radiation among cloud conditions should be interpreted with caution. Bennartz et al. (2013) found that increased surface temperatures during summer 2012 over the GrIS could be partly attributed to thin, low-level liquid clouds. These clouds were thick and low enough to enhance incoming longwave radiation, while at the same time thin enough to allow sufficient incoming shortwave radiation to pass through them. Future model projections indicate increased cloudiness associated with the advection of warm, moist air masses and rainfall over Greenland (Stendel et al. 2007; Collins et al. 2013). The effect on SEB is dependent on timing and surface type; however, current models disagree on the distribution of increased cloudiness over the course of a year (Vihma et al. 2016). More wintertime clouds would have a warming effect through increased incoming longwave radiation across both ice and tundra. While increased summertime cloudiness would have a cooling effect over tundra, it could have a warming effect over GrIS and glaciers (Bennartz et al. 2013). However, increased winter cloudiness may also result in more snowfall shortening the snow-free period.

## Conclusions

Due to the complex, non-linear interactions between temperature, precipitation and cloudiness, modelling current conditions and especially predicting future changes remain a challenge (Vihma et al. 2016). Being able to quantify the spatiotemporal variability in SEB is crucial for improved understanding of the climate system, especially for the feedback mechanisms amplifying anthropogenic warming in the Arctic. This signifies the importance of year-round monitoring and a dense network of measurement stations across surface types. In this study, we have investigated SEB across various surface types in Greenland and assessed factors regulating spatiotemporal variation. Our main conclusions include the following:

- The main differences between tundra and ice sites during summer include large differences in surface temperatures, sensible heat flux having opposite signs, and surface melt being the most important energy sink for ice sites. For tundra energy is used for sensible and latent heat flux warming the atmospheric boundary layer and ground heat flux lead to soil heating and permafrost thaw.
- The end-of-winter snow depth, timing of snow melt and the length of the snow-free season are controlling factors for SEB across surface types. Longer snow-free periods increase melting of glaciers and ice sheet, whereas for tundra, earlier snow melt may promote permafrost thaw, although hydrological changes affecting soil thermal conductivity may modulate thaw rates.
- The impacts of cloudiness on SEB are sensitive to surface type, time of year and cloud characteristics. During winter, clouds limit energy losses through longwave radiation across surface types. During summer, clouds have a cooling effect over tundra and a warming effect over ice surfaces, primarily because of the differences in surface albedo.

## Electronic supplementary material

Below is the link to the electronic supplementary material.

Supplementary material 1 (PDF 976 kb)
