# Supplementary material for: Spatiotemporal variability in surface energy balance across tundra, snow and ice in Greenland
Source: Ambio. 2017 Jan 23;46(Suppl 1):81–93. doi: 10.1007/s13280-016-0867-5 (PMC5258660; doi:10.1007/s13280-016-0867-5)
Supplement: Supplementary file 1 — Supplementary material 1 (PDF 976 kb) [file 13280_2016_867_MOESM1_ESM.pdf]

Ambio

Electronic Supplementary Materials
This supplementary material has not been peer reviewed.

Title: **Spatiotemporal variability in surface energy balance across tundra and ice in Greenland**

Authors: Magnus Lund, Christian Stiegler, Jakob Abermann, Michele Citterio, Birger U. Hansen, Dirk van As

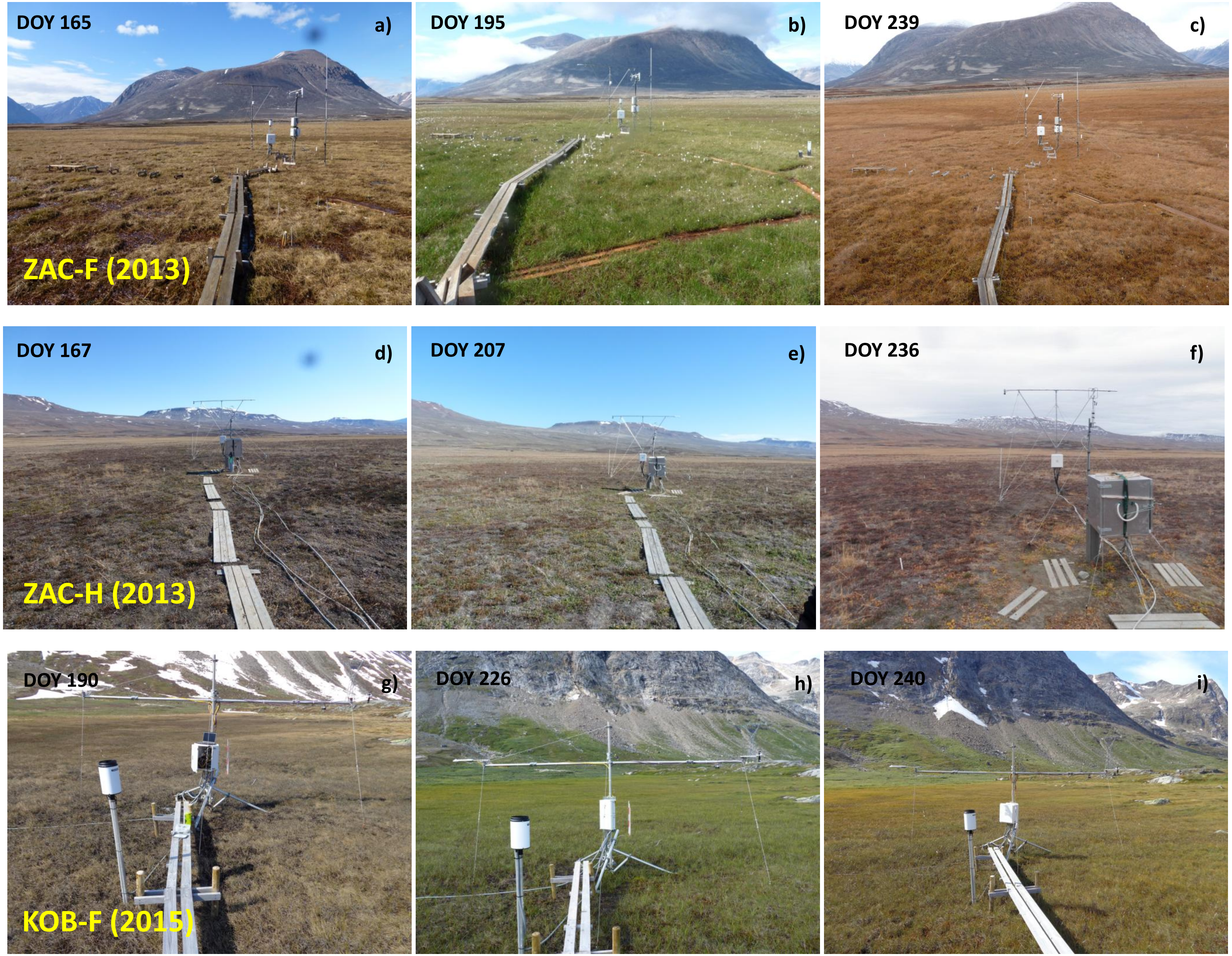
DOY 165
a)
ZAC-F (2013)
DOY 195
b)
DOY 239
c)
DOY 167
d)
ZAC-H (2013)
DOY 207
e)
DOY 236
f)
DOY 190
g)
KOB-F (2015)
DOY 226
h)
DOY 240
i)

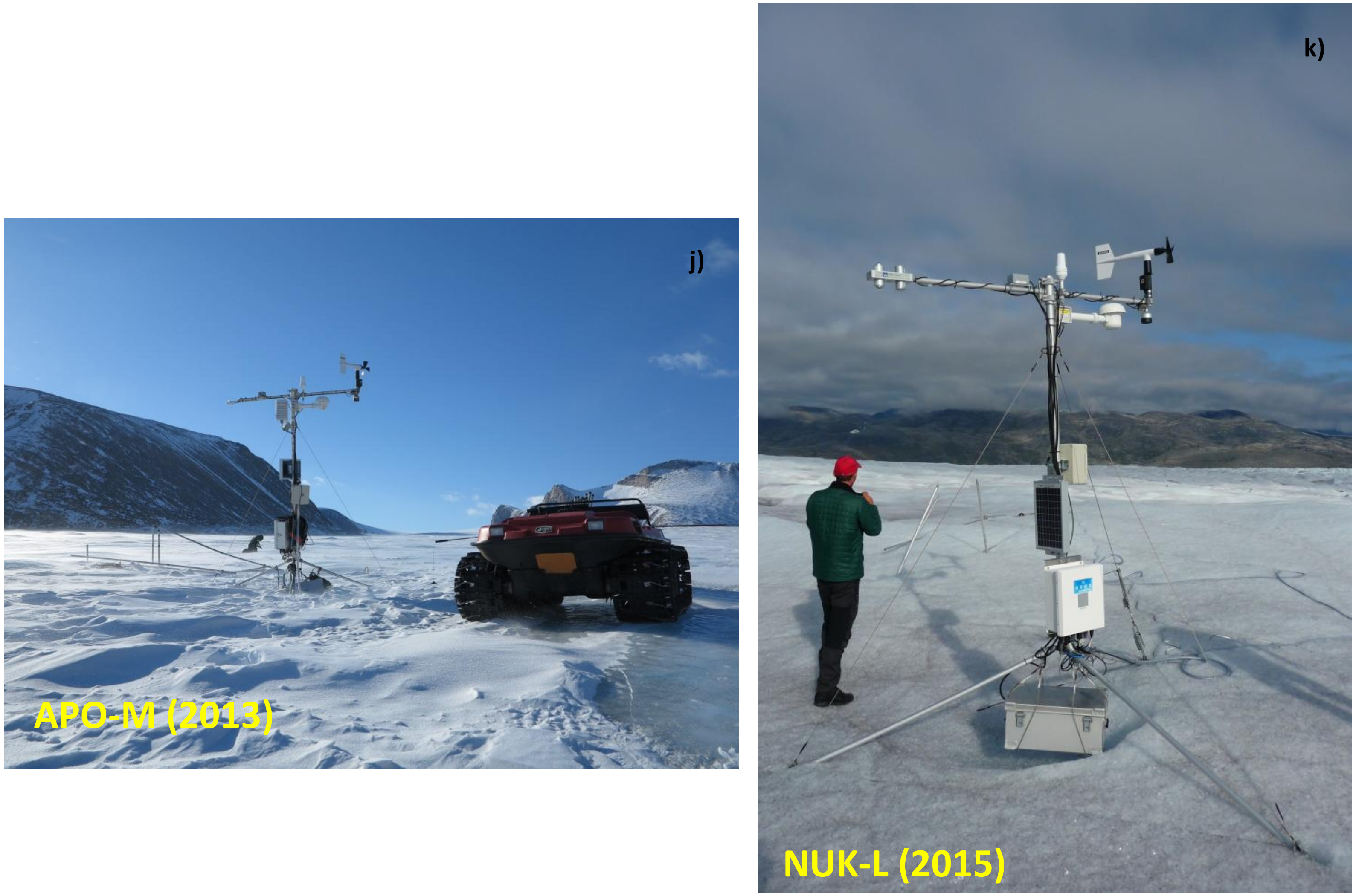

Fig. S1. Photos from each site, a-c) ZAC-F, d-f) ZAC-H, g-i) KOB-F, j) APO-M, k) NUK-L
